# Factors related to liability for damages for adverse events occurring in long-term care facilities

**DOI:** 10.1371/journal.pone.0283332

**Published:** 2023-05-19

**Authors:** Mayumi Tsuji, Hiroki Fukahori, Daisuke Sugiyama, Ardith Doorenbos, Katsumi Nasu, Yuriko Mashida, Hirofumi Ogawara

**Affiliations:** 1 Department of Health Sciences, Nagasaki University Graduate School of Biomedical Sciences, Nagasaki-shi, Nagasaki, Japan; 2 Graduate School of Health Management, Faculty of Nursing and Medical Care, Keio University, Fujisawa-shi, Kanagawa, Japan; 3 Faculty of Nursing and Medical Care, Keio University, Fujisawa-shi, Kanagawa, Japan; 4 Department of Bio-behavioral Health Science, College of Nursing, University of Illinois, Illinois, Chicago, United States of America; 5 Faculty of Nursing, Yasuda Women’s University, Hiroshima-shi, Hiroshima, Japan; 6 Graduate School of Health Care Sciences, Tokyo Medical and Dental University, Tokyo, Japan; University of Florence, ITALY

## Abstract

Globally, residents of long-term care facilities (LTCFs) often experience adverse events (AEs) and corresponding lawsuits that result in suffering among the residents, their families, and the facilities. Hence, we conducted a study to clarify the factors related to the facilities’ liabilities for damages for the AEs that occur at LTCFs in Japan. We analyzed 1,495 AE reports from LTCFs in one Japanese city. A binomial logistic regression analysis was conducted to identify factors associated with liability for damages. The independent variables were classified as: residents, organizations, and social factors. In total, 14% of AEs resulted in the facility being liable for damages. The predictors of liability for damages were as follows: for the resident factors, the increased need for care had an adjusted odds ratio (AOR) of 2.00 and care levels of 2–3; and AOR of 2.48 and care levels of 4–5. The types of injuries, such as bruises, wounds, and fractures, had AORs of 3.16, 2.62, and 2.50, respectively. Regarding the organization factors, the AE time, such as noon or evening, had an AOR of 1.85. If the AE occurred indoors, the AOR was 2.78, and if it occurred during staff care, the AOR was 2.11. For any follow-ups requiring consultation with a doctor, the AOR was 4.70, and for hospitalization, the AOR was 1.76. Regarding the type of LTCF providing medical care in addition to residential care, the AOR was 4.39. Regarding the social factors, the reports filed before 2017 had an AOR of 0.58. The results of the organization factors suggest that liability tends to arise in situations where the residents and their family expect high quality care. Therefore, it is imperative to strengthen organizational factors in such situations to avoid AEs and the resulting liability for damages.

## Introduction

As of 2022, all countries are facing an increasingly aging population. By 2050, one in six people worldwide will be aged 65 years or older [1]. Although some older adults have no health problems, others do. Such problems include those associated with physical functions, comorbidities, and/or cognitive impairments, and in such cases, older adults require specific types of nursing care-based support throughout their daily lives [2–4]. For example, in many countries, people aged 65 years and older receive long-term care (LTC) services either at home or in long-term care facilities (LTCFs) [5, 6].

In Japan, since the introduction of the public long-term care insurance (LTCI) system in April 2000, homecare, and residential services have been provided to Japanese adults aged 65 years and older. Residential services are provided to people requiring personal care or health care. There are facilities in which people can undergo rehabilitation and return home or live there permanently [7]. In Japan, in 2017, for the first time, major newspapers reported the number of deaths related to adverse events (AEs) occurring in Japanese LTCFs [8, 9]. Based on an unofficial government report, 1,547 residents of various LTCFs died, and these deaths were related to AEs associated with aspiration and choking under the care that these residential facilities provide [8, 9]. Although the definition of the term AE in LTCF-related services is yet to be settled in Japan, according to the Conceptual Framework of the International Classification of Patient Safety [10], the World Health Organization used various terms, such as near-miss events, harmless events, or AE. AEs are defined as a harm or injury resulting from medical care [11, 12], including the failure to provide needed care [12].

### Characteristics of AEs

Several countries have conducted studies on AEs occurring in medical and LTCFs. The reported AEs that have occurred in hospitals in the US and Sweden include “falls” [13–15], “errors in medication” [13, 15], and “pressure-related ulcers” [14, 15]. Although some AEs are not serious, some can be life threatening, fatal, or may result in permanent injuries [15].

Similar AEs have also been reported in various LTCFs across Japan. A Japanese national survey examined incidents such as near-miss events and AEs in LTCFs and showed that 90% of LTCFs had AEs that resulted in the LTCF being held liable for damages [16]. Such AEs included falls, aspirations, bedsores, wandering, or errors in medication [16]. It remains unclear whether the types of AEs or injuries are related to liability for damages.

### Liability for damages resulting from AEs

In the UK, US, and Australia, AEs have resulted in increased hospitalizations and lawsuits [17]. In Japan, Canada, Australia, and other Western countries, AEs have resulted in liability for damages associated with negligence among medical staff or care workers [18–20]. In principle, in Japan, liability for damages refers to a person’s or organization’s responsibility to compensate for damages resulting from intentional negligence to the body or property of another person, in accordance with the provisions of the Civil Code of Japan [21]. Some Japanese facilities have been held liable for AEs, such as falls or aspiration [22, 23], and for fall events resulting from nursing staff regarded as negligent [24]. In Japan, lawsuits resulting from aspiration are more common in LTCFs than in hospitals, and damages have been awarded within a range of four to 40 million yen in such lawsuits [25]. The payment of high-cost liability for damages resulting from AEs, such as falls and aspiration, is a financial burden to the facilities and the individual staff involved.

Without a careful analysis of AEs and the resulting liabilities, it is difficult to establish solutions aimed at preventing or limiting liability. The quality of medical care [26, 27] and post-accident response [28] have been reported as aspects that influence liability. However, it is yet to be verified whether the type of AE or the type of injury is associated with liability. From the perspective of crisis management, we lack the necessary information to minimize the risks of secondary damage, such as relationship problems, poor quality of care, lawsuits, terminations, turnover of staff, and bankruptcy.

### Relationship between LTC and liability in Japan

Globally, Japan has the highest proportion of an aging population [29]. Additionally owing to the historical shift to nuclear families, it is expected that in the near future, many older adults who are highly dependent on medical care will live in LTCFs. Residential care used to be managed by local governments, but after the start of the LTCI system in 2000, this changed to contracts with facilities. LTCFs that provide services under the banner of safety and security may face increasing levels of liability and lawsuits in the future. There have been 85 nursing care accident-related court cases in Japan (between 2011 and early January 2016) in which falls, aspiration, wandering, and bedsores have resulted in lawsuits [30].

Therefore, LTCFs require measures for ensuring residents’ safety to improve residents’ quality of life and avoid liability resulting from lawsuits associated with AEs. The causes of liability for damages are associated with residents’ conditions, the quality of care in LTCFs, and staffing. Most of the AEs resulting in liability for damages occur within facilities in cases where the facility structures are inadequate, such as when a care worker delays calling the resident for emergency transport or where safety measures were not implemented when a resident chose to spend time alone [30, 31]. For example, in a death by choking case in a fee-based home for older adults, Resident A was served a bread roll in a private room. The staff did not put a nurse call by Resident A’s hand. The staff then left for approximately 20 minutes after serving the food. Resident A had a history of refractory reflux esophagitis and hiatal hernia of the esophagus, and prior to moving into the facility, Resident A had eaten a porridge diet and had sometimes vomited. Resident A’s doctor provided this information to the facility. The facility was found to have failed to take appropriate measures to prevent aspiration and to be in breach of its duty of care [32]. Liability also occurs when actions by LTCF staff, such as nurses and care workers, affect nursing residents directly [31, 33].

### Factors of liability for damages

**Fig 1:**
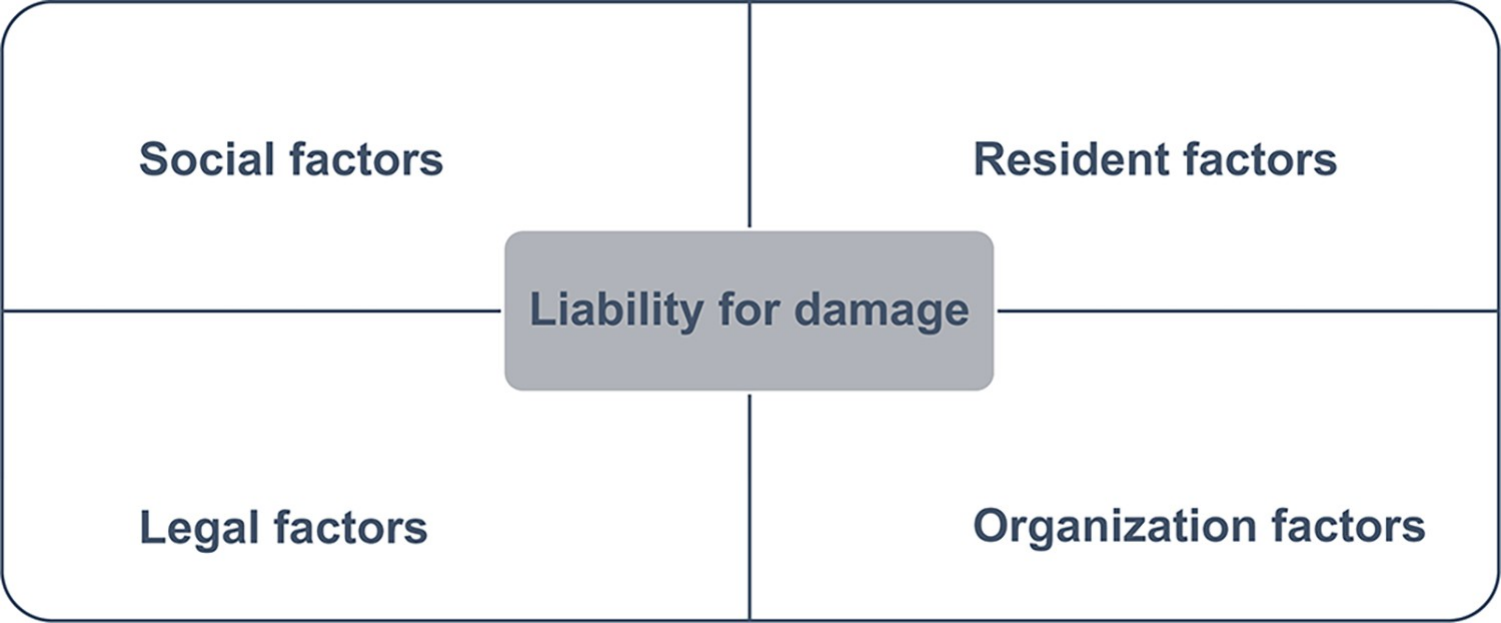
The conceptual framework of factors for liability for damages.

Liability for damages can be caused by a multitude of factors. From our perspective, these can be classified into four factors (Fig 1). The resident factors include physical condition and type of injury; organization factors include facility type, response after AE, staff level, and work environment: social factors include public opinion and policies for older adults; and legal factors include competency of attorney. Analyzing the situations that have already resulted in liability for damages will help identify risk factors. If the predictors of liability for damages can be identified, further physical, mental, and financial damages to not only residents and their caregivers but also medical staff and care workers can be avoided.

### Purpose of this study

To the best of our knowledge, no previous studies have focused on the relationship between specific AEs in Japan and subsequent liability for damages. This study thus aimed to (1) clarify the characteristics of AEs among residents in LTCFs and (2) explore factors related to liability for damages for AEs occurring in LTCFs.

## Materials and methods

### Design

This was a retrospective study of existing data using descriptive statistical analysis.

### Setting

We gathered information of reported AEs submitted by a LTCI service delivery facility in a single city in Japan. These AEs covered approximately 10 years of data, from December 2011 to February 2020. In 2011, the city had a population of 66,932 people aged 65 years or older who received LTCI services. In 2020, 78,104 people received LTCI services. That year, the proportion of the population aged 65 years or above in this city (31.7%) was higher than that in Japan overall (28.8%) or worldwide (9.3%) [34]. In this city, 84 LTCI facilities were operating in 2011, and 34 new facilities opened afterward, resulting in 118 LTCFs. The total number of LTC beds was 3,787 during the last reported data collected in early 2020.

### LTCFs in Japan

In Japan, based on the Ministerial Ordinance, facilities are required to report promptly to the local government any AEs occurring during the provision of LTCI services that involve a facility resident. The study targeted LTCFs where LTCI services are available in the facility 24 hours a day. These included intensive care homes for older adults, long-term care health facilities (LTCHF), group homes for dementia patients, and fee-based homes for older adults. The characteristics of these LTCF services vary according to the government’s purpose or the intended target population. LTCHFs are long-term care and rehabilitation facilities aimed at discharging the residents back home; intensive care homes are permanent residences for residents who are stable but require regular long-term care; group homes for dementia patients are small-scale and homelike accommodation for residents with mild to moderate dementia; fee-based homes for older adults are congregate housing [7, 35, 36].

Specifically, LTCHFs are required to have one physician and one functional trainer who performs rehabilitation, such as a physical therapist, occupational therapist, or speech-language pathologist, per 100 residents as part of the facility staffing standards. A residential facility for older adults is usually a place to live, not a place to receive medical treatment. Older adults who are highly dependent on medical care live in LTCHFs instead, which are considered midlevel between hospitals and non-LTCHFs [37, 38].

### Sample

AE reports in this city included injuries and deaths, infectious diseases, food poisoning, staff violating laws, and natural disasters. We excluded natural disasters and reports of staff violating laws from the data analyzed as this information was not consistent with the study’s purpose.

**Fig 2:**
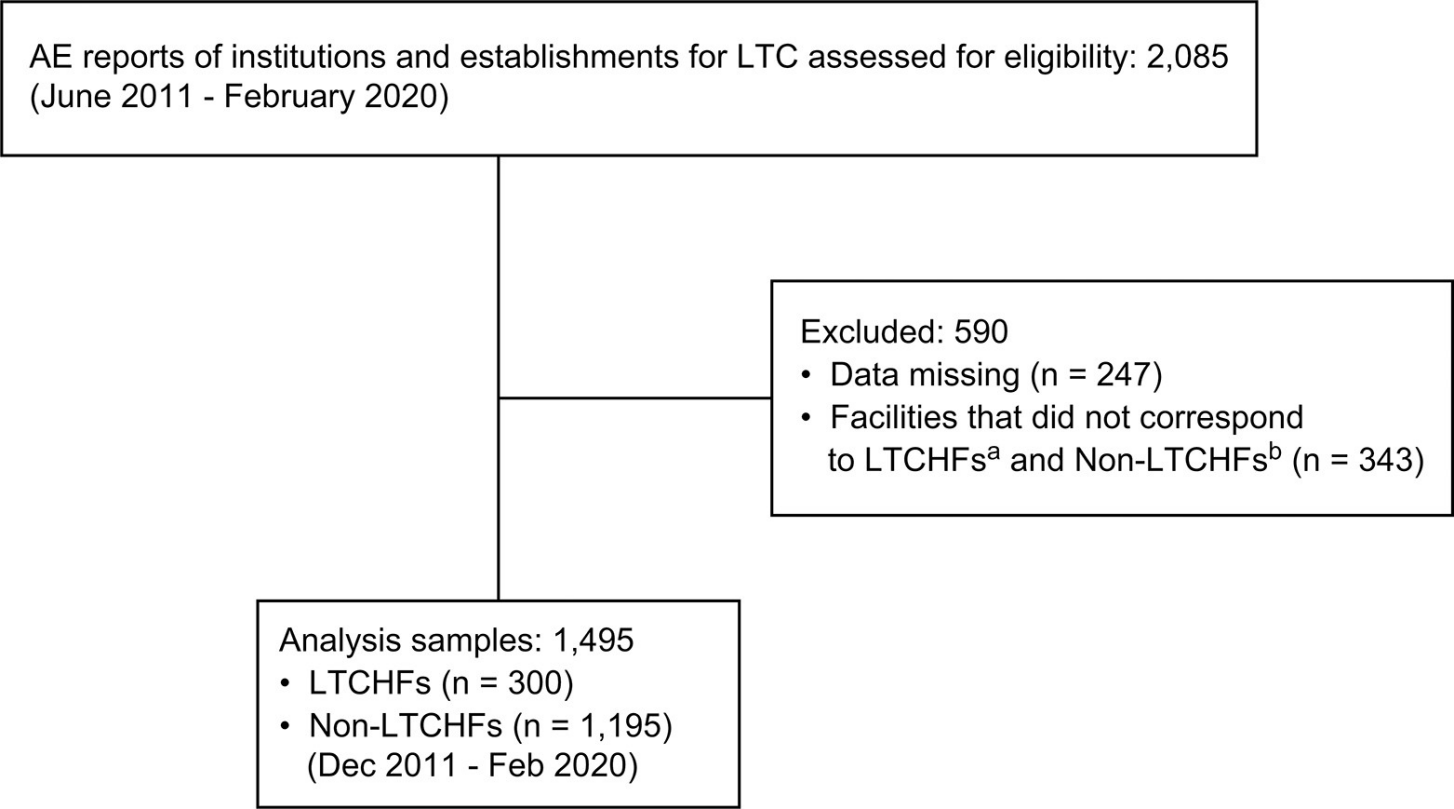
Process for selecting LTC AE reports. ^a^ LTCHFs, long-term care health facility (these facilities have a legal obligation to employ a physician). ^b^ Non-LTCHFs include intensive care homes for older adults, group homes for dementia patients, and fee-based homes for older adults.

Fig 2 shows the process of determining the study’s inclusion and exclusion criteria as well as the number of targets for the analysis of AE reports. We excluded AE reports with missing data and facilities that were not 24-hour LTCFs. In the end, we analyzed 1,495 incident reports, of which 300 were from LTCHFs and 1,195 from non-LTCHFs.

### Data collection

We requested AE reports data from the local government; access is permitted based on local government ordinances when a citizen requests disclosure [39]. Because AE reports contain sensitive information that can help identify individuals and facilities, only de-identified data are provided. We obtained the requested data after receiving a notice of decision for partial disclosure, according to which any data that identified individuals or facilities had been deleted by the local government.

### Measures

The variables were classified into three categories, as shown in Fig 1. The resident factors included information on the age, sex assigned at birth, LTC requirement certification, type of the injury (e.g., bruises, wounds, fractures, death, or others, which included sprains, dislocation, and pneumonia), the response to the AE (e.g., consultation with a doctor, hospitalization needed, and surgery needed). Organization factors included information on the type of facility for each resident who experienced the AE, and the circumstances of the AE included time and location, type of AE (e.g., falls, unexplained injury, medication error, aspiration, or others, which included infectious disease and wandering), and the facility’s liability for damages. Social factors included information on AE reports every year. We determined that there was no variable for legal factors in our dataset.

In the data, we stratified the residents’ age groups into Q1 (59–83 years old), Q2 (84–88 years old), Q3 (89–92 years old), and Q4 (93–106 years old). The span of the report years was divided into two parts by the median year. Residents’ LTC requirement certification was also stratified into three groups: care levels ≤1, 2–3, and 4–5, where a higher number indicated the need for more support. The LTCI system in Japan provides services according to a person’s certification at one of seven levels (support levels 1–2 and care levels 1–5), which depend on disease condition and functional ability. LTCI care levels require more care than LTCI support levels, and the higher the level, the greater the care required. Examples of LTCI certification levels include support level 1: “Requiring some support in instrumental activities of daily living but independent in basic activities of daily living,” care level 1: “requiring partial assistance in at least one basic task of activities of daily living,” and care level 5: “requiring care in all tasks of activities of daily living” [7, 40, 41].

The “response and results of the AE” data provided was free text. From that data, we created new items, such as “AEs during care,” “consultation with a doctor,” “hospitalization needed,” and “surgery needed.”

### Data analysis

Descriptive statistics were calculated for demographic data (i.e., attributes of residents and facilities) and circumstances of the AE (e.g., time and location, occurrence during staff care [yes/no], type of AE, type of injury, response to the AE, and liability). Pearson’s chi-square test was computed to compare AEs that occurred in LTCHFs versus those that occurred in non-LTCHFs.

Binomial logistic regression analysis was conducted to identify the factors associated with AEs that resulted in liability for damages. The dependent variable was the presence or absence of liability for damages. The independent variables were resident factors (resident age, sex, LTC requirement certification, type of injury, response after the AE [whether consultation with a doctor, hospitalization, or surgery was needed]), organization factors (AE occurrence time and location, occurrence during staff care, the type of AE, and whether the AE occurred in a LTCHF or a non-LTCHF), and social factors (AE report year [before or after the median year]).

In the binomial logistic regression model of liability for damages, there were 23 independent variables, including dummy variables. The established rule of 10 events per variable [42] implied that the appropriate number of events for the dependent variable was 230 more. Hosmer-Lemeshow goodness of fit tests, Cox-Snell R^2^, and Nagelkerke R^2^ were used to evaluate the models. Statistical analysis was performed using SPSS version 27 (IBM Corp., Armonk, NY, USA). All reported probabilities (p-values) were two-sided, and p-values < .05 were considered statistically significant.

### Ethical considerations

Because the data provided by the city did not disclose any identifying information about the individuals or facilities, informed consent was not required in this study. This study was approved by the ethics committee of Keio University Graduate School of Health Management (reference number 2020–21) and Nagasaki University Graduate School of Biomedical Sciences (reference number 21030403). This study was conducted in accordance with the Declaration of Helsinki (World Medical Association 2013).

## Results

A total of 1,495 AE reports submitted to the municipality by LTCFs between 2nd December 2011 and 8th February 2020 were analyzed.

### AEs that occurred in LTCFs

Table 1 shows the characteristics of the residents and LTCFs in the AE reports on LTCFs. Regarding the demographics of the residents who experienced AEs, the mean age was 87.8 (SD 7.2, range 59–106) years, and 82.7% were female. Three-quarters (76.3%; n = 1,140) of the residents had been assigned an LTC requirement certification care level 2 or higher. Almost two-thirds of the AEs (60.6%; n = 906) were reported after 2017 (median, range 2011–2020). AEs occurred most frequently between 6:00 a.m. and 11:59 a.m. (34.0%, n = 508), followed by the period from 12:00 p.m. to 5:59 p.m. (28.6%, n = 428). Moreover, 16.8% (n = 251) of the AEs occurred during staff care. Most of the AEs were falls (72.6%, n = 1,086), whereas others included unexplained injuries (9.8%, n = 147), medication errors (3.9%, n = 58), and aspiration (1.9%, n = 29). Most of the injuries included fractures (52.4%, n = 783), wounds (15.7%, n = 235), bruises (15.4%, n = 230), and death (2.2%, n = 33). The facility was held liable for damages owing to the AE in 13.6% of the reports (n = 203).

**Table 1:** Characteristics of the residents and LTCFs in the AE reports (N = 1,495).

		*n*	%
**Age** ^a^,	59–83	351	(23.5)
	84–88	375	(25.1)
	89–92	372	(24.8)
	93–106	397	(26.6)
**Sex**	Female	1236	(82.7)
**LTC requirement certification**	Care level ≤ 1	355	(23.7)
	Care levels 2–3	635	(42.5)
	Care levels 4–5	505	(33.8)
**Report year**	< 2017	589	(39.4)
	≥ 2017	906	(60.6)
**AE time**	0:00 a.m.–5:59 a.m.	240	(16.1)
	6:00 a.m.–11:59 a.m.	508	(34.0)
	12:00 p.m.–5:59 p.m.	428	(28.6)
	6:00 p.m.–11:59 p.m.	319	(21.3)
**AE location**	Indoors	1414	(94.6)
	Outdoors	81	(5.4)
**AE during care**	Yes	251	(16.8)
	No	1244	(83.2)
**Type of AE**	Fall	1086	(72.6)
	Unexplained injury	147	(9.8)
	Medication error	58	(3.9)
	Aspiration	29	(1.9)
	Other ^b^	176	(11.8)
**Type of injury**	Bruises	230	(15.4)
	Wounds	235	(15.7)
	Fractures	783	(52.4)
	Death	33	(2.2)
	Other ^c^	214	(14.3)
**Consultation with a doctor**	Yes	1396	(93.4)
	No	99	(6.6)
**Hospitalization needed**	Yes	562	(37.6)
	No	933	(62.4)
**Surgery needed**	Yes	385	(25.8)
	No	1110	(74.2)
**Liability for damages**	Yes	203	(13.6)
	No	1292	(86.4)
**LTCHF**	Yes	300	(20.1)
	No	1195	(80.0)

Some facilities and/or residents may be represented in more than one report.

LTC, long-term care; LTCF, long-term care facility (any type); LTCHF, long-term care health facility (these facilities have a legal obligation to employ a physician)

^a^ Age was adjusted by 59–83 years old, 84–88 years old, 89–92 years old, 93–106 years old.

^b^ Other AE types include infectious disease, wandering, pica, etc.

^c^ Other injury severities include sprains, dislocations, pneumonia, etc.

### AEs in LTCHFs compared with non-LTCHFs

Table 2 shows the characteristics of AEs that occurred in LTCHFs compared with those that occurred in non-LTCHFs. Significant differences were found in AE type, injury type, hospitalization, surgery, and liability for damages (p = .005, p < .001, p < .001, p = .004, and p < .001, respectively).

**Table 2:** Comparison of characteristics of AEs in LTCHFs and Non-LTCHFs (*N* = 1,495).

	LTCHFs			non-LTCHFs		*P*-value
	*n*		%	*n*	%	
**Age** ^**a**^,	59–83	79	(26.3)	272	(22.8)	.11
	84–88	79	(26.3)	296	(24.8)	
	89–92	67	(22.3)	305	(25.5)	
	93–106	75	(25.0)	322	(26.9)	
**Sex**	Female	248	(82.7)	988	(82.7)	1.00
**LTC requirement certification**	Care level ≤1	60	(20.0)	295	(24.7)	.22
	Care levels 2–3	136	(45.3)	499	(41.8)	
	Care levels 4–5	104	(34.7)	401	(33.6)	
**Report year**	< 2017	122	(20.7)	467	(79.3)	.62
	≥ 2017	178	(19.6)	728	(80.4)	
**AE time**	0:00 a.m.–5:59 a.m.	59	(19.7)	181	(15.1)	.07
	6:00 a.m. –11:59 a.m.	88	(29.3)	420	(35.1)	
	12:00 p.m. –5:59 p.m.	81	(27.0)	347	(29.0)	
	6:00 p.m. –11:59 p.m.	72	(24.0)	247	(20.7)	
**AE location**	Indoors	289	(96.3)	1125	(94.1)	.13
	Outdoors	11	(3.7)	70	(5.9)	
**AE during care**	Yes	41	(13.7)	210	(17.6)	.11
	No	259	(86.3)	985	(82.4)	
**Type of AE**	Fall	239	(79.7)	847	(70.9)	**.005**
	Unexplained injury	29	(9.7)	118	(9.9)	
	Medication error	4	(1.3)	54	(4.5)	
	Aspiration	2	(0.7)	26	(2.2)	
	Other ^b^	26	(8.7)	150	(12.6)	
**Type of injury**	Bruise	55	(18.3)	175	(14.6)	**< .001**
	Wound	38	(12.7)	197	(16.5)	
	Fracture	178	(59.3)	605	(50.6)	
	Death	5	(1.7)	28	(2.3)	
	Other ^c^	24	(8.0)	190	(15.9)	
**Consultation with a doctor**	Yes	286	(95.3)	1110	(92.9)	.13
	No	14	(4.7)	85	(7.1)	
**Hospitalization needed**	Yes	142	(47.3)	420	(35.1)	**< .001**
	No	158	(52.7)	775	(64.9)	
**Surgery needed**	Yes	97	(32.3)	288	(24.1)	**.004**
	No	203	(67.7)	907	(75.9)	
**Liability for damages**	Yes	92	(30.7)	111	(9.3)	**< .001**
	No	208	(69.3)	1084	(90.7)	

Age was analyzed using the Mann-Whitney U test, and the other variables were analyzed using Pearson’s chi-square test. Some facilities and/or residents may be represented in more than one report.

LTCHFs: long-term care health facility (these facilities have a legal obligation to employ a physician); Non-LTCHF include intensive care homes for older adults, group homes for dementia patients, and fee-based homes for older adults.

^a^ Age was adjusted by 59–83 years old, 84–88 years old, 89–92 years old, 93–106 years old.

^b^ Other AE types included infectious diseases, wandering, pica, etc.

^c^ Other injury types included sprains, dislocations, pneumonia, etc.

### Factors related to AEs in LTCFs

Table 3 shows the predictors of the facility’s liability for damages, obtained using binomial logistic regression analysis. The Hosmer–Lemeshow test results indicated the absence of statistical significance (goodness of fit χ^2^ = 11.642, p = 0.168). Additionally, the Cox-Snell R^2^ and Nagelkerke R^2^ values were 0.107 and 0.195, respectively. The following AE characteristics were all significant factors of liability for damages: occurring among residents with care levels 2–3 and 4–5; in the report year before 2017; AEs occurring between 12:00 p.m. and 5:59 p.m.; AEs that occurred indoors and during staff care; with the types of injuries being bruises, wounds, or fractures; those that required consultation with a doctor or hospitalization; and those that occurred in LTCHFs.

**Table 3:** Predictors of AEs leading to liability for damages (*N* = 1,495).

	OR	95% CI	*P*-value	AOR	95% CI	*P*-value
**Resident factors**							
**Age, Q1**	Ref					
**Q2**	1.20	[0.86, 1.67]	.16	1.30	[0.82, 2.05]	.27
**Q3**	0.98	[0.70, 1.39]	.93	1.19	[0.74, 1.91]	.48
**Q4**	0.76	[0.53, 1.08]	.13	0.86	[0.53, 1.39]	.53
**Sex, Female**	0.90	[0.61, 1.31]	.57	0.84	[0.55, 1.30]	.44
**LTC requirement certification, Care level ≤ 1**	Ref			Ref		
**Care levels 2–3**	2.18	[1.37, 3.47]	< .001	2.00	[1.23, 3.26]	**.005**
**Care levels 4–5**	2.79	[1.75, 4.44]	< .001	2.48	[1.48, 4.14]	**< .001**
**Type of injury, Bruise**	1.17	[0.79, 1.74]	.43	3.16	[1.40, 7.14]	**.006**
**Wound**	1.09	[0.73, 1.63]	.67	2.62	[1.21, 5.65]	**.01**
**Fracture**	1.38	[1.02,1.86]	.04	2.50	[1.19,5.27]	**.02**
**Death**	0.20	[0.03,1.43]	.11	0.54	[0.06,5.16]	.60
**Consultation with a doctor, Yes**	5.35	[1.68,17.05]	.005	4.70	[1.33,16.64]	**.02**
**Hospitalization needed, Yes**	1.59	[1.18, 2.14]	.002	1.76	[1.14, 2.72]	**.01**
**Surgery needed, Yes**	1.18	[0.85, 1.64]	.32	0.75	[0.49,1. 17]	.21
**Organization factors**							
**AE time, 0:00 a.m.–5:59 a.m.**	Ref					
**6:00 a.m.–11:59 a.m.**	1.35	[0.83, 2.20]	.22	1.52	[0.90, 2.57]	.12
**12:00 p.m.–17:59 p.m.**	1.65	[1.02, 2.69]	.04	1.85	[1.09, 3.14]	**.02**
**18:00 p.m.–23:59 p.m.**	1.23	[0.73, 2.10]	.44	1.36	[0.78, 2.39]	.28
**AE location, Indoors**	2.48	[0.99, 6.19]	.05	2.78	[1.05, 7.35]	**.04**
**AE during care, Yes**	1.72	[1.20, 2.45]	.003	2.11	[1.30, 3.43]	**.002**
**Type of AE, Fall**	1.11	[0.79, 1.55]	.55	0.75	[0.40, 1.41]	.38
**Aspiration**	0.23	[0.03, 1.72]	.15	0.54	[0.06, 5.30]	.60
**Unexplained injury**	1.14	[0.70, 1.83]	.61	0.91	[0.41, 1.99]	.81
**LTCHF, Yes**	4.32	[3.16, 5.91]	< .001	4.39	[3.14, 6.14]	**< .001**
**Social factors**						
**Report year, ≥ 2017**	.60	[0.45, 0.81]	< .001	0.58	[0.41, 0.80]	**.001**

OR, odds ratio; CI, confidence interval; AOR, adjusted odds ratio estimated by multivariable analysis; Q1, 59–83 years old; Q2, 84–88 years old; Q3, 89–92 years old; Q4, 93–106 years old; LTC, long-term care; LTCHF, long-term care health facility (these facilities have a legal obligation to employ a physician).

### Factors related to AEs in LTCHFs compared to non-LTCHFs

Table 4 shows the binomial logistic regression analysis of predictors of liability for damages from AEs that occurred in LTCHFs versus non-LTCHFs. The Hosmer–Lemeshow test results indicated the absence of statistical significance for the models for LTCHF’s liability for damages and non-LTCHF’s liability for damages (goodness of fit χ^2^ = 10.416, p = 0.237; χ^2^ = 9.198, p = 0.326). Additionally, the Cox-Snell R^2^ and Nagelkerke R^2^ values of the model for LTCHF’s liability for damages were 0.119 and 0.169, respectively. Additionally, the Cox-Snell R^2^ and Nagelkerke R^2^ values of the model for non-LTCHF’s liability for damages were 0.066 and 0.143, respectively. In LTCHFs, AEs occurring among residents at care levels 2–3 and during times of day between 12:00 p.m. and 5:59 p.m. or 6:00 p.m. and 11:59 p.m., or in the report year before 2017 were significant predictors of liability for damages.

**Table 4:** Predictors leading to liability for damages in LTCHFs compared with Non-LTCHFs (*N* = 1,495).

	LTCHFs			Non-LTCHFs		
	AOR	95% CI	*P*-value	AOR	95%CI	*P*-value
**Resident factors**						
**Age, Q1**	Ref			Ref		
**Q2**	1.01	[0.47, 2.16]	.98	1.49	[0.81, 2.74]	.20
**Q3**	0.90	[0.40, 2.02]	.79	1.45	[0.79, 2.68]	.23
**Q4**	0.92	[0.42, 2.03]	.85	0.80	[0.42, 1.52]	.49
**Sex, Female**	0.48	[0.25, 0.99]	.05	1.41	[0.75, 2.64]	.28
**LTC requirement certification, care level ≤1**	Ref			Ref		
**care levels 2–3**	2.50	[1.15, 5.43]	**.02**	1.88	[0.98, 3.60]	.06
**care levels 4–5**	1.96	[0.83, 4.64]	.12	2.79	[1.44, 5.43]	**.003**
**Type of injury,**						
**Bruise**	2.13	[0.61, 7.48]	.24	9.14	[2.97, 28.10]	**< .001**
**Wound**	1.95	[0.57, 6.67]	.29	6.12	[2.20, 17.03]	**< .001**
**Fracture**	1.00	[0.32, 3.10]	1.00	10.38	[3.69, 29.20]	**< .001**
**Hospitalization needed, yes**	1.85	[0.85, 4.05]	.12	1.95	[1.14, 3.34]	**.01**
**Surgery needed, yes**	1.04	[0.50, 2.16]	.90	0.53	[0.30, 0.94]	**.03**
**Organization factors**						
**AE time, 0:00 a.m.–5:59 a.m.**	Ref					
**6:00 a.m.–11:59 a.m.**	2.22	[0.91, 5.45]	.08	1.07	[0.57, 2.03]	.83
**12:00 p.m.–17:59 p.m.**	3.63	[1.50, 8.83]	**.004**	1.16	[0.60, 2.22]	.66
**18:00 p.m.–23:59 p.m.**	3.42	[1.38, 8.46]	**.008**	0.60	[0.28, 1.29]	.19
**AE location, indoors**	5.09	[0.58, 44.87]	.14	2.00	[0.68, 5.88]	.21
**AE during care, yes**	1.97	[0.75, 5.18]	.17	2.36	[1.33, 4.19]	**.003**
**Type of AE,**						
**falls**	1.58	[0.50, 5.99]	.47	0.52	[0.25, 1.11]	.09
**Unexplained injury**	1.83	[0.42, 8.34]	.43	0.74	[0.29, 1.89]	.53
**Social factors**						
**Report year, ≥ 2017**	0.55	[0.31, 0.97]	**.04**	0.58	[0.38, 0.89]	**.01**

In non-LTCHFs, significant factors of liability for damages were residents with care levels 4–5; AEs that occurred during staff care; injury types of bruises, wounds, or fractures; and the need for hospitalization.

## Discussion

This study revealed the characteristics of AEs reported from LTCFs in a city in Japan. Additionally, this study examined the frequency of the liability for damages and associated factors related to the liability. To the best of our knowledge, this study is the first to investigate the factors associated with liability for damages in LTCFs in Japan.

### Characteristics of AEs reported

In this study, falls accounted for 70% of all AEs and was the most likely type of AE to occur. This finding is similar to that of previous reports from the US and Sweden [14, 15]. Fractures accounted for more than half of the injuries observed in this study. This is understandable as the mean age of the patients was 87.75 years, and most of them were females. Older people lose bone mass with age and are more prone to fractures, while the number of trabeculae is reduced more among females after menopause than among males [43, 44]. However, fractures increase a person’s care level and decrease quality of life, which makes fall prevention and strengthening measures important in LTCFs. Intervention studies have investigated LTCF residents’ walking conditions [45], the use of assistive devices [46], and the setup of room environments that match individual needs and fall prevention exercises [47]. Physical and educational interventions that enable residents to walk safely are also important.

### Frequency of liability for damages

We found that only 203 cases (14%) of AEs occurring in the 118 LTCFs in one Japanese city resulted in liability for damages.

This result seems to be extremely low compared to what we expected, given that nursing homes in the US experienced claim litigation every two years on average [48]. This study could be indicating that the rhetoric regarding this burden of liability in Japan is overblown, although there was a period of public uproar because a Japanese newspaper reported that 1,547 deaths that occurred in 2017 resulted from incidents in LTCFs [8, 9]. The number of complaints and litigation is expected to continue to increase as LTCI service users and their families become increasingly aware of safety and security. It is desirable for residents, families, facilities, and staff to avoid AEs resulting in litigation as much as possible. The LTCF is naturally liable for compensation if the occurrence of AEs is due to negligence. However, according to the study of medical disputes, the frequency of positive compensation in non-negligent cases was significantly higher if miscommunication (e.g., insufficiently informed consent, insufficient explanation of the event, and little response after the event) was perceived [49]. Patients and families also expect a positive dialogue with hospitals even after compensation. Among their expectations, they want to know information about safety improvement efforts in response to the AE [50, 51]. We believe that risk communication is important and that similar problems can occur in the LTC field owing to communication errors. It is necessary to accumulate research not only on AE prevention but also on post-AE response.

### Factors associated with liability for damages

We found that AEs during the day and AEs that occurred during staff care resulted in liability for damages. This study suggested the need to strengthen organizational systems to ensure that there are no situations that could easily result in negligence regarding staffing and resident care systems as well as the residential environment.

### Resident factors

In the resident category, having a care level higher than two was a significant risk factor for AEs resulting in liability for damages. The higher the level of care, the more the resident and family would expect quality of care because the facility and staff are more involved in the resident’s care. Previous studies have reported that patients with high instrumental activities of daily living dependency and comorbidities have a higher incidence of AEs [52]. Thus, with an increase in care level, more attention should be paid to avoid AEs and to ensure accountability.

This study found that some variables in the type of injury were significant correlates of liability for damages. In Japan, care workers and nurses account for 65.3% and 10.4% of LTCF staff, respectively. Therefore, non-medical workers dominate the workplace [53]. This could be a problem with proper treatment and response to injuries after AEs occur.

In addition, in the subgroup analyses, non-LTCHFs with more non-medical staff than LTCHFs had more variables for the type of injury. Compared with LTCHFs, non-LTCHFs have fewer medical professionals and are not legally obliged to have physicians on duty because residents do not require medical care management. The facility management systems are important for investigating and strengthening, including the understanding of residents’ health statuses and response after AEs. What victims of AEs want most is a sincere response from their medical and welfare service providers and an understanding of the suffering they have undergone [28]. From a crisis management perspective, research has been conducted on communication strategies, such as errors in disclosure and apologies [50, 51, 54–57]. However, those who provide medical and other types of care to residents of LTCFs must work toward fundamental solutions to the quality of care rather than damage payments and claim solutions. Nurses, as health care professionals, must work toward enhancing collaboration with non-medical staff to protect the health of residents and to conduct better communication.

### Organization factors

In the organization category, AE occurrence time, particularly from midday to evening, and AEs that occurred indoors were significant predictors of liability for damages. Previous studies have reported that incidents and AEs tend to occur in the morning or from nighttime to early morning, when there are fewer staff members [58, 59]. Therefore, we predicted that AEs occurring at times when there is a low staff number are more likely to result in liability for damages. However, LTCFs took liability for damages owing to AEs occurring during the daytime and indoors, when staffing is relatively more adequate than at nighttime or when outdoors. We believe that residents and their families expect a higher quality of care during daytime than nighttime or early morning when residents are asleep, and staffing is expected to be lower. The best-quality nursing homes had litigation risks that are lower than their low-quality counterparts [27]. In LTCFs’ quality, this includes not only the frequency of AEs and quality of care level but also staffing. Owing to Japan’s increase in the population of adults aged 65 years or older, there is currently a shortage of approximately 260,000 workers in Japanese LTCFs [60]. In light of these considerations, we must investigate whether there are any circumstances regarding staffing levels, the system of resident care, or the residential living environment that could easily lead to negligence.

In this study, we found that the risk of liability was significantly higher for AEs that occurred during staff care. It is difficult for residents and their families to accept physical injuries, such as bruises and fractures, and the associated cost of medical treatment, such as doctor visits and hospitalization, resulting from AEs that are caused by staff care. AEs that occur during staff care sometimes result in lawsuits [31, 33]. In these lawsuits, staff including nurses and care workers are often pursued the responsibility of such AEs. In these cases, the obligation of security and duty of care of the LTCF professions (i.e., nurses or care workers) often become the rationale for the judge’s decision [38]. This makes it understandable that they are more likely to be liable for compensation. Nantsupawat (2022) reported that poor nurse staffing is associated with missed care, and missed care is associated with AEs and lower quality of care [61]. As this study includes some cases in which falls occurred while a staff member was assisting other residents (no details of AE are shown in this result), it is also necessary to investigate the circumstances (e.g., resident physical condition, information about staff, and work environment) at the time the AE occurred. These circumstances are not included in the data of our study.

LTCHFs were significant predictors of liability for damages. In Japan, LTCHFs are intermediate facilities for people leaving the hospital and hoping to return home. Therefore, the residents are in the subacute to convalescent phase. It has been reported that the majority of LTCHF residents have dementia, central nervous system diseases, cardiac and cardiovascular diseases, or musculoskeletal diseases, with some residents experiencing acute exacerbations, such as falls, high fevers, and aspiration pneumonia [62]. If a resident experiences an AE, their condition could change easily. Additionally, their response after AEs occur could be inappropriate because they are cared for mainly by non-medical staff who dominate the workplace in Japanese LTCFs.

Type of AE was not a significant risk factor for liability for damages. We know that AEs, including falls, aspiration, wandering, and pressure ulcers, have led to lawsuits [31]. Falls and aspiration, which have been reported to have a higher award rate than other AEs, have sometimes been awarded more than $20 million in damages [31, 38] Aspiration is directly related to life-threatening conditions, such as choking and pneumonia, especially for residents with impaired feeding and swallowing functions. In this study, the relation to liability for damages was not the AE type but the injuries and the situation arising from an AE. We believe that analyzing each AE is important to take measures.

### Social factors

In the social category, the report year being before 2017 was a significant risk factor for liability for damages. In 2015, a famous case was reported in Japan in which a nurse at an intensive care home for older adults made a mistake in delivering a snack and was charged with manslaughter by a causal link to a resident’s choking [33]. Some facilities have refrained from serving some snacks since the accident [63]. As of 2018, in a Japanese LTCFs survey, more than 90% of LTCFs enrolled in liability insurance and have established a claim handling system [16]. In this study, AEs reported after 2017 were significantly less likely to be liable for damages. The impact of the news and court reports could have promoted the establishment of organizational management systems owing to heightened awareness of resident safety in many LTCFs.

In the end, to the best of our knowledge, this is the first study to identify risk factors related to liability for damages in the LTC setting. In this study, organizational factors, such as time of the AEs, AEs occurring during staff care, and response to AEs, were associated with liability for damages, thereby suggesting the need to strengthen measures in organizational aspects from a crisis management perspective.

In addition, various injuries were associated with liability for damages, thereby suggesting the need for stronger collaboration between nurses and case workers in the LTC setting, where many non-medical staff are involved in the response after an AE.

### Limitations

This study has some limitations. First, there is a generalizability problem. Because the facilities included in the analysis were localized to one western Japanese city, sample bias may have occurred. Second, measurements related to the liability for damages should be improved in future studies. In our data, the data about liability itself was limited to only the presence or absence of liability for damages. Additionally, because we had no information on the development of lawsuits over time or the amount of compensation, we could not assess the institutional aspects of the cases. Data about the type of injury, for example, a “wound” in the data available to us could be a minor or major wound and vary in the degree of cures available. We were unable to assess the relationship between the severity of injury and liability. Third, the goodness of fit of the model in the binomial logistic regression analysis showed the absence of statistical significance in the Hosmer-Lemeshow test, but the Cox-Snell R^2^ and Nagelkerke R^2^ values suggest that other factors could be associated with liability for damages, such as legal factors, and social factors (only report year in our data) not included in this data.

## Conclusion

This study revealed the factors associated with liability for damages for AEs occurring in LTCFs. The risk of liability for damages was significantly high when AEs occurred at daytime or in staff care, and when injuries occurred that required additional care. Organizational efforts, such as staffing, staff levels, and post-AE response are necessary to avoid AEs and the liability for damages. Among the various factors involved in liability for damages, the results of organization factors suggest that the liability tends to arise in situations where the residents and their family expect high quality care.

## References

[1] United Nations Department of Economic and Social Affairs Population Division. World population prospects 2019: ten key findings. [Internet]. [cited 2023 Jan 20]. Available from: https://population.un.org/wpp/Publications/Files/WPP2019_10KeyFindings.pdf

[2] Iecovich E. The long-term care insurance law in Israel: present and future. J Aging Soc Policy. 2012;24:77–92. doi: 10.1080/08959420.2012.628892 22239283

[3] Kim H, Kwon S, Yoon NH, Hyun KR. Utilization of long-term care services under the public long-term care insurance program in Korea: implications of a subsidy policy. Health Policy. 2013 Jul;111:166–174. doi: 10.1016/j.healthpol.2013.04.009 23706386

[4] Oliver D, Foot C, Humphries R. Making our health and care systems fit for an ageing population. London: The King’s Fund; 2014.

[5] Muir T. Measuring social protection for long-term care. OECD Health Working Paper No. 93. Paris: OECD Publishing; 2017.

[6] Ministry of Health, Labour and Welfare. [Internet]. The situation surrounding the long-term care field. 2020 [cited 2023 Jan 20]. Available from: https://www.mhlw.go.jp/content/12300000/000608284.pdf (in Japanese).

[7] Ministry of Health, Labor and Welfare. [Internet]. Long-term care insurance system of Japan 2016. [cited 2023 Jan 20]. Available from: https://www.mhlw.go.jp/english/policy/care-welfare/care-welfare-elderly/dl/ltcisj_e.pdf (in Japanese).

[8] Yomiuri Shimbun. 1547 people died in accidents in FY17. Yomiuri Database Service. [Internet]. 2019 May 15 [cited 2023 Jan 20]. Available from: https://database-yomiuri-co-jp.kras1.lib.keio.ac.jp/rekishikan/ (in Japanese).

[9] Asahi Shimbun. 1,547 accidental deaths in long-term care facilities, first survey by MHLW in FY2017. Kikuzo II Visual for Libraries. [Internet]. 2019 May 16 [Cited 2023 Jan. 20]. Available from: http://database.asahi.com.kras1.lib.keio.ac.jp/library2/main/top.php (in Japanese).

[10] World Health Organization. [Internet]. Chapter 3. Key concepts and preferred terms. 2009 [cited 2023 Jan 20]. Available from: https://www.who.int/patientsafety/taxonomy/icps_chapter3.pdf.

[11] Brennan TA, Leape LL, Laird NM, Hebert L, Localio AR, Lawthers AG, et al. Incidence of adverse events and negligence in hospitalized patients. Results of the Harvard Medical Practice Study I. N Engl J Med. 1991;324:370–376. doi: 10.1056/NEJM199102073240604 1987460

[12] Levinson DR. Adverse events in skilled nursing facilities: National incidence among Medicare beneficiaries. Washington (DC): US Department of Health and Human Services, Office of the Inspector General; 2014 Feb. Report No.: OEI-06-11-00370.

[13] Andersson Å, Frank C, Willman AM, Sandman PO, Hansebo G. Factors contributing to serious adverse events in nursing homes. J Clin Nurs. 2018;27:e354–e362. doi: 10.1111/jocn.13914 28618102

[14] Iyer P. Liability in the care of the elderly. J Obstet Gynecol Neonatal Nurs. 2004;33:124–131. doi: 10.1177/0884217503261132 14971561

[15] Kapoor A, Field T, Handler S, Fisher K, Saphirak C, Crawford S, et al. Adverse events in long-term care residents transitioning from hospital back to nursing home. JAMA Intern Med. 2019;179:1254–1261. doi: 10.1001/jamainternmed.2019.2005 31329223 PMC6646976

[16] Ministry of Health, Labour and Welfare. [Internet]. Survey and research project on the state of safety and hygiene management systems in welfare facilities for the elderly requiring long-term care. 2019 [cited 2023 Jan 20]. Available from: https://www.mhlw.go.jp/content/12601000/000500363.pdf (in Japanese).

[17] Donaldson L. An organization with a memory. Report of an expert group on learning from adverse events in the National Health Service. London, UK: The Stationery Office, Department of Health; 2000.

[18] Studdert DM, Mello MM, Brennan TA. Medical malpractice. N Engl J Med. 2004;350:283–292. doi: 10.1056/NEJMhpr035470 14724310

[19] Wick MR, Foucar E, Allen PW, Alves VA, Bjornsson J, Bosman F, et al. Medicolegal liability in pathology: an international perspective. Semin Diagn Pathol. 2007;24:65–76. doi: 10.1053/j.semdp.2007.03.007 17633348

[20] Cheluvappa R, Selvendran S. Medical negligence—key cases and application of legislation. Ann Med Surg (Lond). 2020;57:205–211. doi: 10.1016/j.amsu.2020.07.017 32793340 PMC7413923

[21] Matsushita Y, Sugiyama Y, Kobayashi M. Medical safety. 4th ed. Osaka: Medica; 2021. (in Japanese).

[22] Yokota H. Problems of cooperation between care service and medical service for the elderly person judging from lawsuits in the care facility. Bull Soc Med. 2013;30:31–38. (in Japanese).

[23] Hamasaki T, Hagihara A. Medical malpractice litigation related to choking accidents in older people in Japan. Gerodontology. 2021;38:104–112. doi: 10.1111/ger.12506 33169853

[24] Nakamura N, Takaoka C, Nishimura R, Yamashita Y. Judicial precedents concerning falls in hospitals. Med Bull Fukuoka Univ. 2011;38:47–52. (in Japanese).

[25] Inouchi T, Kanezaki H, Kobayashi H. Analysis of precedents for aspiration accidents. J Hosp Gen Med. 2020;16:346–353. (in Japanese).

[26] Harrington C, Stockton J, Hooper S. The effects of regulation and litigation on a large for-profit nursing home chain. J Health Polit Policy Law. 2014;39(4):781–809. doi: 10.1215/03616878-2743039 24842973

[27] Studdert DM, Spittal MJ, Mello MM, O’Malley AJ, Stevenson DG. Relationship between quality of care and negligence litigation in nursing homes. N Engl J Med. 2011;364(13):1243–50. doi: 10.1056/NEJMsa1009336 . 21449787

[28] Vincent C, Young M, Phillips A. Why do people sue doctors? A study of patients and relatives taking legal action. Lancet. 1994 Jun 25;343(8913):1609–13. doi: 10.1016/s0140-6736(94)93062-7 7911925

[29] Statistics Bureau of Japan. Statics topic No. 129. Statistics on the older adult in Japan: in the context of respect-for-senior-citizens day. Available from: https://www.stat.go.jp/data/topics/topi1291.html (in Japanese).

[30] Sato N, Akazawa K, Mitadera Y, Suzuki T, Ibe N, Kirose Y. Clarifying problems with emergency healthcare systems in Japanese long-term care facilities for older people. Health. 2017; 9:1159–1175. doi: 10.4236/health.2017.98084

[31] Misaka A, Hino M, Yamada H, Ishikawa K, Nakamaru T, Tsujimoto C, et al. Various issues related to claims for damages for accidents involving older people in medical and long-term care facilities. Hanrei Times, No. 1425. 2016 (in Japanese).

[32] Case of an appeal for damages. Osaka High Court Decision on February 15, 2013. Case No. 2012 (ネ) 1537, Westlaw Japan. Included in case law database. (in Japanese).

[33] Case of professional negligence resulting in death. the Nagano District Court Decision on March 25, 2019. Case No. 2014 (ワ) 260, Westlaw Japan. Included in case law database. (in Japanese).

[34] Ministry of Health, Labour and Welfare. [Internet]. Annual report on the ageing society (whole version) 2. International trends in aging. 2021. [cited 2023 Jan 20]. Available from: https://www8.cao.go.jp/kourei/whitepaper/w-2021/zenbun/pdf/1s1s_02.pdf (in Japanese).

[35] Nakanishi M, Hattori K, Nakashima T, Sawamura K. Health care and personal care needs among residents in nursing homes, group homes, and congregate housing in Japan: why does transition occur, and where can the frail elderly establish a permanent residence? J Am Med Dir Assoc. 2014 Jan;15(1):76.e1-6. doi: 10.1016/j.jamda.2013.07.006 23981788

[36] Nasu K, Sato K, Fukahori H. Rebuilding and guiding a care community: a grounded theory of end-of-life nursing care practice in long-term care settings. J Adv Nurs. 2020 Apr;76(4):1009–1018. doi: 10.1111/jan.14294 31845377

[37] Ministry of Health, Labour and Welfare. [Internet]. Kaigo roujin hoken shisetu. [cited 2022 Dec 29]. Available from: https://www.mhlw.go.jp/file/05-Shingikai-12601000-Seisakutoukatsukan-Sanjikanshitsu_Shakaihoshoutantou/0000174012.pdf (in Japanese).

[38] Sato T. Kaigo jikocare ni yoru songai baishou seikyu sosyou no saiban rei gaikan. Kashitu anzen hairyo gimu ihan no handan wo chusin to sjite. Hanrei Times No. 1425. 2016. (in Japanese).

[39] Ministry of Internal affairs and communications. [Internet]. Gyousei kikan no hoyuu suru jyouhou no koukai ni kansuru houritsu. J E Gov (Japan). 2009 [cited 2023 Jan 20]. Available from: https://elaws.e-gov.go.jp/document?lawid=411AC0000000042 (in Japanese).

[40] Ministry of Health, Labour and Welfare. [Internet]. Structure of the long-term care requirements certification in the long-term care insurance system. 2015 [cited 2023 Jan 20]. Available from: https://www.mhlw.go.jp/topics/kaigo/kentou/15kourei/sankou3.html (in Japanese).

[41] Ito K, Kawai H, Tsuruta H, Obuchi S. Predicting incidence of long-term care insurance certification in Japan with the Kihon Checklist for frailty screening tool: analysis of local government survey data. BMC Geriatr. 2021;21:22. doi: 10.1186/s12877-020-01968-z 33413151 PMC7792049

[42] Peduzzi P, Concato J, Kemper E, Holford TR, Feinstein AR. A simulation study of the number of events per variable in logistic regression analysis. J Clin Epidemiol. 1996;49:1373–1379. doi: 10.1016/s0895-4356(96)00236-3 8970487

[43] Medical Advisory Secretariat. Prevention of falls and fall-related injuries in community-dwelling seniors: an evidence-based analysis. Ont Health Technol Assess Ser. 2008;8: 1–78. PMC3377567 23074507

[44] Farr JN, Khosla S. Skeletal changes through the lifespan—from growth to senescence. Nat Rev Endocrinol. 2015;11:513–521. doi: 10.1038/nrendo.2015.89 26032105 PMC4822419

[45] Craig JJ, Bruetsch AP, Huisinga JM. Coordination of trunk and foot acceleration during gait is affected by walking velocity and fall history in elderly adults. Aging Clin Exp Res. 2019;31:943–950. doi: 10.1007/s40520-018-1036-4 30194680 PMC7309343

[46] Thies SB, Russell R, Al-Ani A, Belet T, Bates A, Costamagna E, et al. An investigation of the effects of walking frame height and width on walking stability. Gait Posture. 2020;82:248–253. doi: 10.1016/j.gaitpost.2020.09.017 32987344

[47] Gschwind YJ, Kressig RW, Lacroix A, Muehlbauer T, Pfenninger B, Granacher U. A best practice fall prevention exercise program to improve balance, strength / power, and psychosocial health in older adults: study protocol for a randomized controlled trial. BMC Geriatr. 2013;13:105. doi: 10.1186/1471-2318-13-105 24106864 PMC3852637

[48] Stevenson DG, Spittal MJ, Studdert DM. Does litigation increase or decrease health care quality?: a national study of negligence claims against nursing homes. Med Care. 2013 May;51(5):430–6. doi: 10.1097/MLR.0b013e3182881ccc 23552438 PMC4825676

[49] Aoki N, Uda K, Ohta S, Kiuchi T, Fukui T. Impact of miscommunication in medical dispute cases in Japan. Int J Qual Health Care. 2008 Oct;20(5):358–62. doi: 10.1093/intqhc/mzn028 18635588

[50] Moore J, Bismark M, Mello MM. Patients’ experiences with communication-and-resolution programs after medical injury. JAMA Intern Med. 2017 Nov 1;177(11):1595–1603. doi: 10.1001/jamainternmed.2017.4002 29052704 PMC5710270

[51] O’Connor E, Coates HM, Yardley IE, Wu AW. Disclosure of patient safety incidents: a comprehensive review. Int J Qual Health Care. 2010 Oct;22(5):371–9. doi: 10.1093/intqhc/mzq042 20709703

[52] Sears NA, Blais R, Spinks M, Paré M, Baker GR. Associations between patient factors and adverse events in the home care setting: a secondary data analysis of two Canadian adverse event studies. BMC Health Serv Res. 2017;17:400. doi: 10.1186/s12913-017-2351-8 28606073 PMC5469013

[53] Japan Federation of Medical Worker’s Unions. [Internet]. Kaigo roudou jittai chousa houkokusyo. [cited 2023 Jan 20]. Available from: http://irouren.or.jp/research/0ab7e9f308c3af76bdbb2cfce6b32ad4813d1d0a.pdf (in Japanese).

[54] Murtagh L, Gallagher TH, Andrew P, Mello MM. Disclosure-and-resolution programs that include generous compensation offers may prompt a complex patient response. Health Aff (Millwood). 2012 Dec;31(12):2681–9. doi: 10.1377/hlthaff.2012.0185 23213152

[55] Mazor KM, Reed GW, Yood RA, Fischer MA, Baril J, Gurwitz JH. Disclosure of medical errors: what factors influence how patients respond? J Gen Intern Med. 2006 Jul;21(7): 704–10. doi: 10.1111/j.1525-1497.2006.00465.x 16808770 PMC1924693

[56] Choi EY, Pyo J, Lee W, Jang SG, Park YK, Ock M, et al. Perception gaps of disclosure of patient safety incidents between nurses and the general public in Korea. J Patient Saf. 2021 Dec 1;17(8):e971–e975. doi: 10.1097/PTS.0000000000000781 32910040 PMC8612886

[57] Ock M, Choi EY, Jo MW, Lee SI. General public’s attitudes toward disclosure of patient safety incidents in Korea: results of disclosure of patient safety incidents survey I. J Patient Saf. 2020 Mar;16(1): 84–89. doi: 10.1097/PTS.0000000000000428 32106177 PMC7046138

[58] McCloskey R, Donovan C, Donovan A. Linking incidents in long-term care facilities to worker activities. Workplace Health Saf. 2017;65:457–466. doi: 10.1177/2165079916680366 28368696

[59] Arai Y. A consideration of the realities of care accidents: a study of care accident of K city in Osaka. Care Welf. 2009;16:59–65. (in Japanese).

[60] Ministry of Health, Labor and Welfare. [Internet]. Securing of human resources for long-term care and innovation of long-term care situations. 2019 [cited 2023 Jan 20]. Available from: https://www.mhlw.go.jp/content/12300000/000698872.pdf

[61] Nantsupawat A, Poghosyan L, Wichaikhum OA, Kunaviktikul W, Fang Y, Kueakomoldej S, et al. Nurse staffing, missed care, quality of care and adverse events: a cross-sectional study. J Nurs Manag. 2022 Mar;30(2):447–454. doi: 10.1111/jonm.13501 Epub 2021 Nov 26. 34719833 PMC9017335

[62] Japan association of Geriatric Health Services Facility. [Internet]. Report on a research project on medical care for patients with basic diseases and complications in long-term care health facilities. [cited 2023 Jan 20]. Available from: https://www.roken.or.jp/wp/wp-content/uploads/2021/10/21_kisoshikkan.pdf

[63] Yamashita T, Yamazaki K. Balancing service quality and risk management in a nursing-care facility: improving user satisfaction and creating a positive work environment. Japan Marketing Academy. 2022;3(1);62–70. doi: 10.7222/marketingreview.2022.008 (in Japanese).

